# The complex representation and contradicting results linking sexual orientation to allostatic load

**DOI:** 10.1016/j.ssmph.2025.101789

**Published:** 2025-03-20

**Authors:** Gabriel Desjardins, Névéna Chuntova, Robert-Paul Juster

**Affiliations:** a Center on Sex∗Gender, Allostasis, and Resilience (CESAR), Quebec, Canada; b Research Center of the Montreal Mental Health University Institute (CRIUSMM), Quebec, Canada; c Department of Psychology, University of Montreal, Quebec, Canada; d Department of Psychiatry & Addiction, University of Montreal, Quebec, Canada

## Abstract

This commentary discusses a publication by Katsuya Oi and Amanda M. Pollitt using the National Longitudinal Study of Adolescent to Adult Health to assess presumed sexual orientation effects on allostatic load, the ‘wear and tear’ of chronic stress. Their findings indicate that discordant heterosexual women—those whose sexual attractions or behaviors do not align with their heterosexual identity—experience notably higher allostatic load compared to other sub-groups. In contrast, women who identify as non-heterosexual did not exhibit significantly elevated allostatic load. Several theoretical problems, interpretative inadequacies, issues with terminology, and misrepresentation of the existing literature limit the full impact of this original work. In the spirit of collegial critique, the objective of this commentary is to offer potential resolutions and considerations for future research among sexually diverse as well as gender diverse populations.

## 1 Introduction

**This commentary focuses on a study by Oi and Pollitt** in *Social Science and Medicine – Population Health* titled ''The roles of non-heterosexuality outside of identity and gender non-conformity in allostatic load among young adults”. Oi and Pollitt's study employed the National Longitudinal Study of Adolescent to Adult Health (ADD HEALTH) to evaluate allostatic load (AL) levels among individuals identifying as lesbian women, gay men, and bisexual (LGB) in comparison to heterosexual people. The analysis specifically hones in on heterosexual people experiencing non-heterosexual sexual attractions or behaviors (named discordant heterosexuals in Oi and Pollitt's article). The outcomes indicate an absence of AL elevation among men and women who self-identify as non-heterosexual. However, women who self-identified as heterosexuals with non-heterosexual sexual attractions or behaviors manifested a notably higher AL. Furthermore, women with a more “androgynous appearance” independently exhibited increased AL. These findings suggest broadening research on sexual minorities to include individuals without a specific LGB identity, while recognizing the potential stress linked to their sex assigned at birth.

Oi and Pollitt's study has several methodological and theoretical strengths. This study employs a robust longitudinal design with ample statistical power. Moreover, ADD HEALTH is a nationally representative sample that allows for comparisons with up to five waves of data (*N* = 8353). By accounting for multiple covariates, the study also enhances the robustness and reliability of observed findings. Notably, it is the first, to our knowledge, to examine the interplay between Gender Schema Theory, the Minority Stress Model, and AL. This integrative approach enables a comprehensive analysis of the intersections between gender, sex, discrimination, and the biological mechanisms underlying chronic stress.

Despite these strengths, Oi and Pollitt's contribution to this emerging field is not without its issues. In our commentary, we first argue that the study misrepresents key aspects of the existing literature on AL and health disparities among SGD populations. Second, we note that the study perpetuates, to some extent, a linguistically discriminatory stance toward SGD individuals and mostly heterosexual people. Third, while theoretical integration is a notable strength, the study demonstrates an incomplete understanding of the minority stress model and overlooks critical elements of the AL literature. Lastly, despite grounding itself in Gender Schema Theory, we urge caution regarding the accurate and consistent use of the term and theories regarding "androgyny".

### 1.1 Allostatic load and minority stress

Allostasis refers to the body's and the brain's adaptive process of recalibrating physiological systems in response to acute environmental demands (Sterling & Eyer, 1988). While this adaptive process is essential for immediate survival, chronic activation of allostasis can result in AL, which reflects the cumulative "wear and tear" on the body and brain. Over time, sustained allostasis leads to pathophysiological changes, ultimately increasing the risk of disease and death. AL is typically measured through a composite of biomarkers that span multiple systems, including neuroendocrine, immune, metabolic, and cardiovascular systems, all of which are sensitive to the effects of chronic stress (McEwen, 1998).

Research has extensively examined the role of social determinants of health in contributing to AL across diverse populations (Juster). More recently, the AL framework has been applied to understand health disparities within *sexual and gender diverse* (SGD) communities. SGD individuals often face greater health burdens compared to their heterosexual peers, largely due to the chronic stressors associated with marginalization and discrimination (Juster; Mays et al., 2018; Walubita et al., 2021).

Minority stress models posit that health inequalities among SGD people result from unique stressors related to their minority status (es), which go beyond the general stressors experienced by everyone in everyday life (Meyer, 2003). These stressors, both at the individual and societal levels, often accumulate, leading to a higher health burden compared to heterosexual and/or cisgender people. Proximal stressors involve internal psychological processes that are more “subjective”, such as the internalization of societal stigma, negative self-perceptions, and fears of rejection. On the other hand, distal stressors refer to external and more “objective” experiences like discrimination, victimization and social exclusion.

At the systemic level, factors like policies, legislation, and prevailing cultural norms further reinforce stigma, amplifying the stress burden on SGD people. For instance, a recent study showed that structural stigma (indexed according to state-level laws and policies) is linked to elevated AL only in sexually diverse men but not in sexually diverse women (Juster). Likewise, Juster et al. (2024) also found that AL does not differ among women by sexual orientation, suggesting that other forms of structural stigma (e.g., institutionalized gender and structural sexism) might be at play (Homan, 2019). Similarly, another recent study revealed that anti-transgender policies are linked to increased psychological distress in transgender and nonbinary people due to enacted (or “objective”) stigma (Puckett et al., 2024). Overall, the minority stress model accurately illustrates how the interaction of personal, interpersonal, and structural stressors contributes to the observed health disparities in SGD people.

According to the sexual minority stress model and an adapted gender minority stress model (Testa), SGD people encounter unique stressors not experienced by their heterosexual and/or cisgender counterparts (Meyer et al., 2003). Note that while we use the term SGD, stigma related to sexual orientation is not the same as stigma related to gender identity. As hypothesized by our group and others, stress processes associated with SGD identities could explain higher AL among SGD populations. In a large, population-based analysis (*N* = 13,911), Mays et al. (2018) used the National Health and Nutrition Examination Survey (NHANES) to test this hypothesis. Results indicated significant sub-group differences in AL based on sexual orientation, particularly among men. Specifically, bisexual men showed the highest AL, while gay men showed the lowest AL in contrast to heterosexual men. Interestingly, there were no sexual orientation differences in AL for women (Mays et al., 2018), a negative finding also reported by others (Juster; Walubita et al., 2021). Disparities in AL could potentially be explained by differences in health risk behaviors among sexually diverse people, such as substance use, lack of physical activity, overeating, and poor sleep hygiene (Suvarna; Desjardins et al., 2022).

Globally, while their integration of different theories with a robust methodological design offers valuable insights, there is an opportunity to further engage with over a decade's worth of existing literature on sexual orientation and AL, particularly the consistent findings that show a lack of AL differences among women based on sexual orientation. Addressing these gaps could strengthen the study and minimize the potential for biases in the broader scientific literature related to SGD communities and AL. In fact, Oi and Politt found that there were higher AL levels in heterosexual women depending on sexual attraction, and in women depending on gender role expression. Despite problems in terminology that we will discuss next, these findings could have been integrated with current findings on AL among women. Oi and Politt omitted the use of an existing body of literature that could have nuanced their results. These limitations highlight the need for greater alignment with (1) the existing body of knowledge on AL in the context of SGD individuals and (2) the broader theoretical frameworks that explain health disparities in these communities. By commenting on these aspects, we aim to contribute constructively to advancing the field and hope that future research will consider these reflections to further enrich our understanding.

### 1.2 Problems of terminology

A major strength of Oi and Pollitt's (2023) article is the use of the current definition of sexual orientation, based on Laumann et al.'s (1994) work. This foundational research provides a detailed portrait of sexuality, advocating for a multidimensional assessment of sexual orientation. In this framework, sexual orientation is viewed as a composite of three interrelated dimensions: (1) sexual attraction and romance, (2) sexual identity, and (3) sexual behavior. Importantly, this model recognizes fluidity, allowing individuals to move across these continuums over time. Later models have also added romantic attraction, which is overlapping but not identical to sexual attraction (Beaulieu-Prévost & Fortin, 2015; Diamond, 2003).

Notwithstanding, the terminology used in Oi and Pollitt's (2023) study, particularly when referring to self-identified heterosexual people with same-sex attraction, raises some important issues. The analysis does not differentiate between sexual and romantic attraction—a distinction that is gaining recognition in contemporary literature (Beaulieu-Prévost & Fortin, 2015; Diamond, 2003). Although these individuals may not identify with SGD communities, using terms like "discordant" or "deviant" can reinforce pathologizing frameworks that frame same-sex attraction as a deviation from heterosexual norms, from “normality” or “sanity”. Historically, the concept of "normal variation" has challenged this view, positioning all expressions of sexual attraction, whether same-sex or opposite-sex, as natural within the spectrum of human sexuality.

As Drescher (2015) pointed out, outdated terms can be discriminatory, particularly because of their association with the historical pathologization of same-sex attraction. This was evident in the field of mental health when the American Psychological Association removed ego-dystonic homosexuality from the DSM in 1973, shifting away from the pathologizing view of sexually diverse people (Edmiston & Juster, 2022). This change marked a paradigm shift, emphasizing that sexual diversity should be understood as part of normal human variation. Thus, the use of terms like "deviant" or "discordant" can inadvertently revive outdated, homophobic discourses that perpetuate negative attitudes toward SGD individuals within scientific literature.

We acknowledge that some of these terminological issues may stem from the limitations inherent in the dataset used for ADD HEALTH, which may not fully capture the multidimensional nature of sexual orientation and gender nonconformity (GNC). For example, the instruments used in the dataset do not measure sexual orientation and GNC as continuums, which might confound the analysis. As Meyer (2019) suggested, societal norms and attitudes are slow to change. Nevertheless, it is our responsibility within the scientific community to drive this change by adopting forward-thinking and inclusive terminology. In this light, some scholars are even moving away from terms like “minorities” in favor of terms like “diversity” for SGD people to better reflect a less marginalizing perspective and positionality. Similarly, the World Health Organization's shift to using "gender incongruence" for transgender people, instead of pathologizing terms like "gender identity disorder," demonstrates the ongoing evolution of language.

Another problem pertains to the use of the term GNC that we acknowledge can be understood from a diversity of perspectives. For instance, GNC has sometimes been used in the literature to reflect people identifying under the gender nonbinary umbrella, notably when all gender diverse individuals are grouped together in analyses (e.g., “Transgender and Gender Non-Conforming (TGNC)” people). The field of gender research is still emerging, and the terminology continues to evolve, leading to varied and sometimes conceptually distinct uses of terms like GNC. As we already stated, one interpretation pertains to gender identities, as seen in the use of "TGNC" to describe subgroups of participants self-identified as gender diverse in numerous mental health studies. These include research on eating disorders (Bell et al., 2019; Hallward et al., 2023), depression (Borgogna; Chodzen et al., 2019), and anxiety (Borgogna; Butler et al., 2019; Chodzen et al., 2019), among others.

GNC can also be expressed through personality traits. For example, a boy who enjoys expressing emotions openly or a girl who is assertive and independent could be considered GNC according to gender role theories. These expressions may challenge the rigid gender norms that society often associates with one's sex assigned at birth. Indeed, the concept of GNC is primarily concerned with the social and cultural expectations surrounding gender roles and how individuals may not always adhere to these norms. This can be confusing since this definition of GNC does not necessarily correlate with one's gender identity, as it primarily describes outward behaviors or traits that differ from conventional gender expectations.

In other studies, the GNC term can have another meaning, especially when it is used without specifically including transgender identities. In this context, GNC typically refers to individuals whose psychological and behavioral expressions of gender differ from the traditional expectations or norms associated with their sex assigned at birth. This includes a wide range of characteristics such as interests, hobbies, physical appearance, household tasks, and other gendered variables. For example, a person assigned female at birth who enjoys activities traditionally considered "masculine" like playing hockey or engaging in mechanical work, may be seen as GNC. Similarly, an individual who adopts a more "feminine" style of dress or mannerisms, even though they are assigned male at birth, would also be classified in this broad category. It seems that Oi and Politt used GNC partly in line with this definition.

In fact, Oi and Pollitt's article uses the term GNC in a way that seems to conflate it with an "androgyny scale." However, androgyny traditionally refers to the presence of *both* highly masculine and highly feminine personality traits or behaviors, not simply an outward expression of gender (Bem). Moreover, the GNC variable in question primarily measures how participants believe others perceive their appearance, style, and clothing, rather than assessing gender roles or gendered personality traits. This approach reduces GNC to a matter of external observer's perception, which is inherently limiting and overlooks its multidimensional nature.

This view of GNC can be problematic for several reasons. For example, this can exacerbate issues like body image concerns and self-objectification, a process where individuals internalize an external perspective of their body, leading to the self-surveillance of physical appearance. Such issues are particularly prevalent among GNC and gender diverse people (Moradi; Saunders et al., 2024). Simplifying GNC to appearance-based judgments is not only misleading but potentially harmful, as it highlights how others view us rather than allowing for a more nuanced understanding of GNC. While we acknowledge the limitations of the dataset used in the ADD HEALTH, we believe that equating GNC to androgyny is conceptually problematic.

Importantly, reducing GNC to how someone's appearance is perceived or how they dress is discriminatory towards gender diverse and GNC individuals. It would have been more helpful to clarify the limitations of the GNC item upfront, rather than oversimplifying the complex reality of GNC into a one-item Likert scale. Additionally, Oi and Pollitt's study fails to address the potential grouping of transgender individuals among men and women's categories. All women and men are presumed to be cisgender in Oi and Politt's analyses since the ADD HEALTH database lacks questions about transgender experiences. The authors do not acknowledge this limitation. These issues concerning terms and concepts of gender and androgyny could be improved with a greater integration of Gender Schema Theory.

Terminology is closely tied to both scientific progress and social change. This is especially the case when discussing constructs like sexual orientation and gender identity, as seen throughout the history of sexuality research (Kinsey et al., 1948; Laumann et al., 1994). To avoid reinforcing societal stigma against sexual and gender diverse populations in scientific discourse, we recommend using terms like "mostly/predominantly heterosexual" instead of "discordant". In a less concise manner, authors could use “self-identified heterosexuals with same-sex attraction”. Moreover, future research should strive to address the complexity of sexual orientation and gender identity with more precise and validated tools that reflect the nuances of these continuums. Future research should be aligned with current guidelines (Veldhuis et al., 2024) and trends among researchers to avoid any confusion or mistakes that could impede on the public perception of SGD people that is closely tied to their rights being currently in debate in the United States (Kline et al., 2022).

### 1.3 Use of minority stress model

Oi and Pollitt (2023) use Meyer's minority stress model to explore health disparities in the SGD community. However, the study could benefit from a more precise distinction between *distal stressors* (such as prejudice and discrimination) and *proximal stressors* (such as internalized homophobia and concealment). This differentiation is critical for clinicians, particularly when working with individuals who may experience SGD-related feelings or behaviors without identifying as part of the SGD community. The use of proxy variables, like sexual attraction or GNC and androgyny, further blurs the lines between the types of minority stressors, making it difficult to gain a comprehensive understanding of the stressors' unique impacts. It is well-recognized that the use of proxy variables is common in studies involving SGD populations. However, it is crucial for authors to explicitly acknowledge when proxy variables are being used rather than direct measures of complex constructs like androgyny. This transparency helps to minimize misinterpretation of findings and lays the groundwork for more precise and methodologically rigorous research.

As a further recommendation, Hatzenbuehler's (2009) psychological mediation framework could be a pathway towards the use of proxy variables in a more rigorous manner. This framework illustrates how stigma-related stressors affect interpersonal and intrapersonal processes, contributing to health disparities among marginalized populations. This framework is particularly relevant when considering the influence of minority stress on emotional, cognitive, social, and interpersonal aspects of life, all of which contribute to AL. Future research could leverage this framework to facilitate a more nuanced exploration of psychological mechanisms, such as emotion dysregulation, rumination, and rejection sensitivity, which are established mediators between minority stress and health outcomes (Feinstein, 2020). Additionally, both GNC and androgyny could be examined as potential mechanisms within Hatzenbuehler's framework, offering new insights into their roles in the minority stress process. Recent research by Desjardins et al. (2022) adds depth to this discussion by emphasizing the role of rejection sensitivity in stress-related health outcomes. Incorporating these proximal psychological factors into the study of AL could provide greater clarity on the health impacts within SGD and predominantly heterosexual populations. It would allow for a more precise application of Meyer's model, helping to distinguish between proximal stress processes and distal minority stressors.

In summary, while Oi and Pollitt (2023) draw on the minority stress model to investigate health disparities in the SGD community, the lack of clear differentiation between distal and proximal stressors—particularly in terms of stigma exposure—limits the theoretical strength of their application. The reliance on proxy variables such as sexual attraction or GNC androgyny complicates the understanding of minority stress. Future studies would benefit from a clearer delineation of stress processes, particularly proximal ones, as they play a critical role in shaping health outcomes within the framework of Meyer's model.

### 1.4 Representation of existing literature

Oi and Pollitt's (2023) assumptions and interpretations of their findings are based on a limited selection of literature that does not adequately address the complexity of AL in SGD individuals. As stated earlier, studies such as Mays et al. (2018) reveal significant AL differences based on sexual orientation and sex assigned at birth. Oi and Pollitt (2023) omit these findings, despite Mays et al. (2018) using a population-based survey that highlights clear differences in AL among various sexually diverse subgroups. This is important because it challenges the conventional understanding, presenting unique profiles within sexual orientation sub-groups that merit further exploration.

Moreover, Mays et al. (2018) found no AL differences among women based on sexual orientation, suggesting that psychosocial mediation may be at play. This aligns with Juster et al.’s (2013) study, which attributed the low AL in gay men in Quebec to strict dietary practices and physical activity driven by societal pressures towards thinness and musculature. It would have been insightful for Oi and Pollitt to engage with this literature and examine the role of psychosocial factors, such as health behaviors (e.g., smoking, physical activity, sleeping, safe sexual practices), mental health (e.g., disordered eating, anxiety symptoms, borderline personality traits), and societal or cultural factors (e.g., religion or spirituality, community services, social support) providing a comparative framework to better contextualize their findings.

In contrast to Meyer's theory, current literature refutes the uniform application of minority stress across all sub-groups, particularly with bisexual men facing dual stigma that can heighten their AL. Similarly, several studies now show no significant differences in AL among women based on their sexual orientation, emphasizing the role of psychosocial factors like social stigma that must be taken into consideration in future research. These findings highlight the importance of considering both protective and risk factors when assessing AL. Minority stressors resulting from marginalization, alongside sub-group-specific characteristics, likely contribute to these processes (Desjardins et al., 2022). Unfortunately, Oi and Pollitt (2023) do not incorporate this literature into their hypotheses, which could have strengthened their analysis by integrating both biological and psychosocial perspectives.

### 1.5 Inclusivity and intersectionality considerations

Regrettably, the ADD HEALTH dataset, like many surveys incorporating biomarkers, lacks inclusivity with respect to gender diversity. The dataset relies solely on biological sex, defined by the sex assigned at birth (male/female), without accounting for the complexity of gender identity—a continuum that transcends the binary spectrum (Kuyper & Wijsen, 2014). This oversight excludes key SGD populations, particularly transgender and nonbinary individuals, who often experience unique stressors related to stigmatization. Acknowledging this limitation, Oi and Pollitt (2023) should have transparently stated that the data does not encompass gender diverse people and addressed the implications of this gap for their findings. Additionally, proposing potential solutions to enhance inclusivity, such as integrating questions on gender identity in future studies, would have been a valuable contribution.

Although Oi and Pollitt’s (2023) article introduces the concept of androgyny, an important concept in contemporary gender literature, the ADD HEALTH dataset inadequately measures this construct. As stated earlier, the way GNC is assessed reduces a whole community to physical attributes like clothing choices. This method overlooks more profound aspects, such as gender roles, identity, and self-perception. Measuring androgyny through this narrow lens lacks psychometric robustness, oversimplifying the complexity of gender expression. A previous study using Bem's classification of androgyny as high masculine and high feminine gender roles revealed associations to AL that were also omitted in the literature review (Juster). Future research should apply a more comprehensive approach to capture the full spectrum of androgyny (encompassing behaviors, social roles, and personality traits beyond mere appearance), and androgyny should be separated from GNC and transgender identities.

Furthermore, Oi and Pollitt (2023) acknowledge the impact of race and ethnicity on AL, aligning with intersectionality theory (Crenshaw, 2013). However, the study of Walubita et al. (2021), which suggests no AL differences across sexual orientations in Black women, challenges their assumptions. Oi and Pollitt (2023) should have aligned their study more closely with current literature by refining their intersectionality assumptions or adapting their analyses to push the field forward. For example, a more comprehensive intersectional approach, incorporating additional psychosocial factors, could have enriched the contextualization of AL within their study (Desjardins et al., 2022).

## 2 Conclusion

The ADD HEALTH analyses conducted by Oi and Pollitt (2023) provides a distinct perspective from much of the existing literature AL within SGD communities. Their study aligns with Meyer's (2003) minority stress model, which posits that SGD people face unique stressors that contribute to increased AL. However, their findings contrast with several studies that highlight complexities in the interaction between sex assigned at birth and sexual orientation. For example, research by Juster et al. (2013) and Mays et al. (2018) shows that gay men tend to exhibit lower AL than their heterosexual counterparts, a finding that differs from the traditional minority stress narrative. Oi and Pollitt's analysis, however, introduces a different trend by indicating elevated AL among women based on sexual attraction. These findings are relevant and interesting but would have benefited from a more in-depth examination of the current literature in this emergent field.

In fact, a more comprehensive literature review could have further enriched the study by better contextualizing the results within the broader research on sexual orientation, gender identity, and AL. Additionally, incorporating previous recommendations—such as those by Mays et al. (2018)—to include psychosocial variables (e.g., sociodemographics, health behaviors) could provide deeper insights into the varying AL patterns observed across different subgroups. While Oi and Pollitt (2023) did not fully engage with this existing body of literature, their inclusion of androgyny alongside sexual orientation offers a valuable analytical framework; however, ADD HEALTH did not measure this concept thoroughly beyond a single-item. Globally, this approach suggests that considering additional factors beyond sexual orientation alone can yield important insights into health disparities among SGD populations.

## 3 Recommendations

Researchers in the field of sex and gender need to remain attuned to the ever-evolving nature of terminological concepts. It is our responsibility to stay at the forefront of understanding, especially as the SGD population remains a focal point of political discourse. This dynamic societal landscape suggests that terminology, theoretical frameworks, and psychosocial correlates will continue to evolve significantly.

Furthermore, we advocate for moving beyond a dichotomized perspective on gender and sexuality, as such views risk perpetuating discriminatory ideologies. Oppression thrives on division—on defining and separating groups. By embracing the study of gender and sexuality as spectrums, we shift the focus from differences between categorical groups to shared experiences along dimensions. Several frameworks have been suggested, such as Laumann et al. (1994), Kinsey et al. (1948), and Veldhuis et al. (2024). We also suggest the use of Sexual Configurations Theory by Van Anders (2015) that emphasizes the complexity, diversity, and contextual nature of sexuality by considering multiple dimensions and intersections of sexual experiences.

To sum up, if the scientific community collectively rejects binary constructs of gender and sexuality while being careful in the use of theories and terms, we can diminish the relevance of dehumanizing perceptions and foster meaningful systemic changes toward inclusivity and equity. This is particularly important in the wake of increasingly right-wing politics in North America and internationally that tend to be more discriminatory towards SGD populations. As scientists, we must reframe this societal regression in the understanding and acceptance of SGD people. Now more than ever, research should move beyond a deficit-based lens and actively highlight the resilience, strengths, and thriving aspects of SGD communities. By prioritizing strengths-based research, we can illuminate the protective factors and coping mechanisms that foster well-being, offering a more balanced and empowering narrative. The SGD population is rich in strength, diversity, and beauty—focusing solely on challenges risks overshadowing the many ways in which these communities flourish.

Ethical guidelines from Elsevier were followed.

## CRediT authorship contribution statement

**Gabriel Desjardins:** Writing – review & editing, Writing – original draft, Conceptualization. **Névéna Chuntova:** Writing – review & editing. **Robert-Paul Juster:** Writing – review & editing, Supervision, Conceptualization.

## Declaration of competing interest

The authors declare that they have no known competing financial interests or personal relationships that could have appeared to influence the work reported in this paper.

## Data Availability

No data was used for the research described in the article.
